# The Effect of Gymnema Sylvestre on Motivation to Consume Sweet Foods—A Qualitative Investigation

**DOI:** 10.3390/nu17172718

**Published:** 2025-08-22

**Authors:** Imogen Nelson, Rozanne Kruger, David Hsiao, Eric Stice, Ajmol Ali

**Affiliations:** 1 School of Sport, Exercise and Nutrition, Massey University, Auckland 0632, New Zealand; imogennelson@windowslive.com (I.N.); r.kruger@griffith.edu.au (R.K.); chowkapow619@gmail.com (D.H.); 2 School of Health Sciences and Social Work, Griffith University, Gold Coast, QLD 4222, Australia; 3 Department of Psychiatry and Behavioural Sciences, Stanford University, Stanford, CA 94305, USA; estice@stanford.edu

**Keywords:** Gurmar, impulse, sugar-sweetened, behaviour, mindful eating, pleasant

## Abstract

**Background/Objectives:** Excessive intake of sugar-sweetened food (SSF) increases obesity risk. Various psychological, physiological, and environmental factors may drive high consumption of SSF. Due to blocking sweet tastes, the herb Gymnema sylvestre (GS) has been shown to reduce SSF consumption, but its impact on motivation to eat SSF is unknown. This research aimed to qualitatively investigate adults’ perceptions regarding effects of GS on their motivation to eat SSF when administered systematically (three times/day in-between meals, i.e., GS-SYS treatment) or ad libitum (up to six times/day at participants’ discretion, i.e., GS-ADLIB) over 14 days, compared to placebo (taste-matched mint; PLAC-SYS). **Methods:** This study represents the qualitative investigation of a placebo-controlled randomised cross-over trial, conducted as three 14-day phases. The qualitative investigation included interviews at baseline and three post-testing phases. Seven participants (mean age 34.7 ± 13.8 years; two males, five females) agreed to participate. Twenty-eight interviews (across phases) were thematically analysed using NVivo software, identifying themes and highlighting changes in motivation to eat SSFs across the study. **Results:** The GS-SYS and GS-ADLIB treatments made SSFs unpleasant to eat and increased mindful eating, subsequently increasing motivation to avoid SSFs. External factors could increase or decrease motivation, depending on individual circumstances. Participants preferred GS-SYS and GS-ADLIB over PLAC-SYS, feeling it was more effective at changing behaviours related to SSF intake. Self-control over SSF intake changed during the study, mostly due to external factors, and in part GS-ADLIB. **Conclusions**: Participants found both GS administrations successful as motivation to avoid SSF; GS-ADLIB was considered most effective.

## 1. Introduction

High intake of energy-dense foods, including sugar-sweetened foods (SSFs), promote obesity, defined as excess body fat, which is associated with increased morbidity and mortality [1,2]. Daily sugar intake of less than 10% of total energy intake is recommended for decreasing non-communicable disease risk; however, worldwide sugar intake still exceeds these recommendations [3,4]. Scrutiny of sugar-reduction initiatives, including sugar taxes, pharmaceuticals such as appetite suppressants, and dietary sugar restriction, suggests that these initiatives may yield only partial results in reducing motivation to consume SSF [1,5,6,7]. Motivation to eat SSFs may have prehistoric origins, when energy was scarce and sweetness signalled valuable energy-dense foods [8,9,10]. Today’s developed nations rarely face survival-threatening famine, but urbanisation rates exceed the ability of human genomes to adapt concurrently, so preferences for sweet foods persist [10,11]. Motives for eating SSFs can have physiological, psychological, or external origins [12,13,14,15]. Having a ‘sweet tooth’ has been shown to increase pleasure derived from SSF and could increase risks associated with the negative side-effects of high sugar intake [16]. However, whether having a sweet tooth does increase the risk of non-communicable disease development is unknown [17].

The herb Gymnema sylvestre (GS) blocks sweet tastes, with saponins (called Gymnemic acids (GAs)) which attach to sweet taste receptors to prevent sweetness perception [18,19]. Acutely administered GS has been shown to reduce pleasantness of, desire for, and total intake of SSFs [16,20,21,22]. Gastric emptying, glycaemic response, and appetite are not affected by GS [23]. Gymnemic acids have reduced activation of neural reward circuitry in response to anticipated tastes and actual tastes of a high-calorie milkshake [24]. Sugar cravings may be reduced by using GS, highlighting its potential to reduce body weight by decreasing SSF intake [21,25]. In addition, those with a sweet tooth have exhibited larger decreases in pleasantness and desire for SSF with administration of GS, suggesting those with a sweet tooth are particularly sensitive to GS [16,22].

Studies investigating effects of GS on pleasantness of, desire for, total intake of SSF, as well as other outcomes related to SSF consumption have mostly used GS in acute doses, either once, twice, or seven times [16,20,21,23,24,25]. Turner et al. tested the effects of GS intake over 14 days, using a regimented administration of GS, with participants being instructed to take the supplement three times a day [22]. Testing GS over longer periods of time is more conducive to determining the applicability of the herb to settings outside a laboratory and therefore its use in day-to-day life. Turner et al. used GS in a systematic way; however, both the effects of ad libitum consumption and the impact on motivation to consume SSF should be investigated [22]. Ad libitum consumption is of particular interest as it may pose a more appropriate form of GS administration for individuals who want to curb acute sugar cravings. Several behaviour modification studies have already shown that ad libitum interventions may be more effective than a systematic approach, justifying investigation of this in GS administration [26,27].

In addition to the available evidence highlighting that current sugar-reduction initiatives are ineffective, there is also a dearth of evidence regarding the motivations to eat SSF and how GS may affect these motivations [6,7].

The aim of this study was to investigate the effects of GS administered over 14 days, both systematically (i.e., fixed administration three times a day) or ad libitum (i.e., administration up to six times a day at participants’ discretion) relative to a placebo control condition, on the motivation to eat SSF in adults that self-identified as having a sweet tooth. This paper is part of a larger mixed-method study investigating the effect of consuming GS over 14 days on reducing sugar intake; here, we describe the qualitative outcomes of the project.

## 2. Materials and Methods

### 2.1. Participant Screening and Recruitment

This study reports the qualitative investigation of a single blind placebo-controlled randomised cross-over trial, conducted in three 14-day phases [28]. The randomised cross-over trial results have been previously reported [28]. The current manuscript focuses on the qualitative aspect of the study; see Table S1 for a complete checklist of prudent information included in this study related to the research team, study design and analysis. This qualitative investigation employed a mixed-methods approach, grounded in a pragmatist paradigm, and utilised a theme identification methodology to analyse data from open-ended interviews [29]. Interviews were designed with a flexible structure to explore the effects of GS on participants’ desire to consume SSF, changes in total SSF intake, and the associated motivation for consumption. The study adopted a phenomenological approach to examine participants’ lived experiences and the meanings they attached to these changes [29].

Convenience sampling methods were used to select participants. All participants (adult males and females) were screened to have a sweet tooth, not be taking medications affecting smell or taste, and not have any health conditions. Specifically, people with Diabetes Mellitus were excluded as GS may cause hypoglycaemia [30]. An eligibility screener using online software (Qualtrics Experience Management, Provo, UT, USA) determined potential participant eligibility. Sweet tooth status was assessed with the sugar addiction questionnaire and the Dutch eating behaviour questionnaire [31,32]. Eligible participants included those who scored ≥25 percentile in the sugar addiction and Dutch eating behaviour questionnaires, were 18–60 years old (to reduce effects of age-related taste and smell changes), had English as a spoken language, and were able to give written informed consent [33]. The study was conducted between July and October 2022 in the North Shore, Auckland, NZ, USA. Recruitment strategies included posters with scannable QR codes for access to screening tools. These were placed in public areas (e.g., café, library) and posted on social media. From 192 individuals screened, 82 were deemed eligible to take part in the study and 32 were recruited into the trial [28]. This sample size was based on a G*Power (version 3.1.9.7) calculation suggesting approximately 30 participants would enable detection of changes in sweet taste preference, with an allowance for possible dropouts [21]. All recruited participants were invited to participate in the qualitative study. Seven of the recruited participants (aged 22–55 years, mean age of 34.7 ± 13.8 years; two males and five females) accepted the invitation and completed four interviews (at baseline and then after each intervention period). A total of 28 interviews were completed across the study.

### 2.2. Study Design

**Figure 1:**
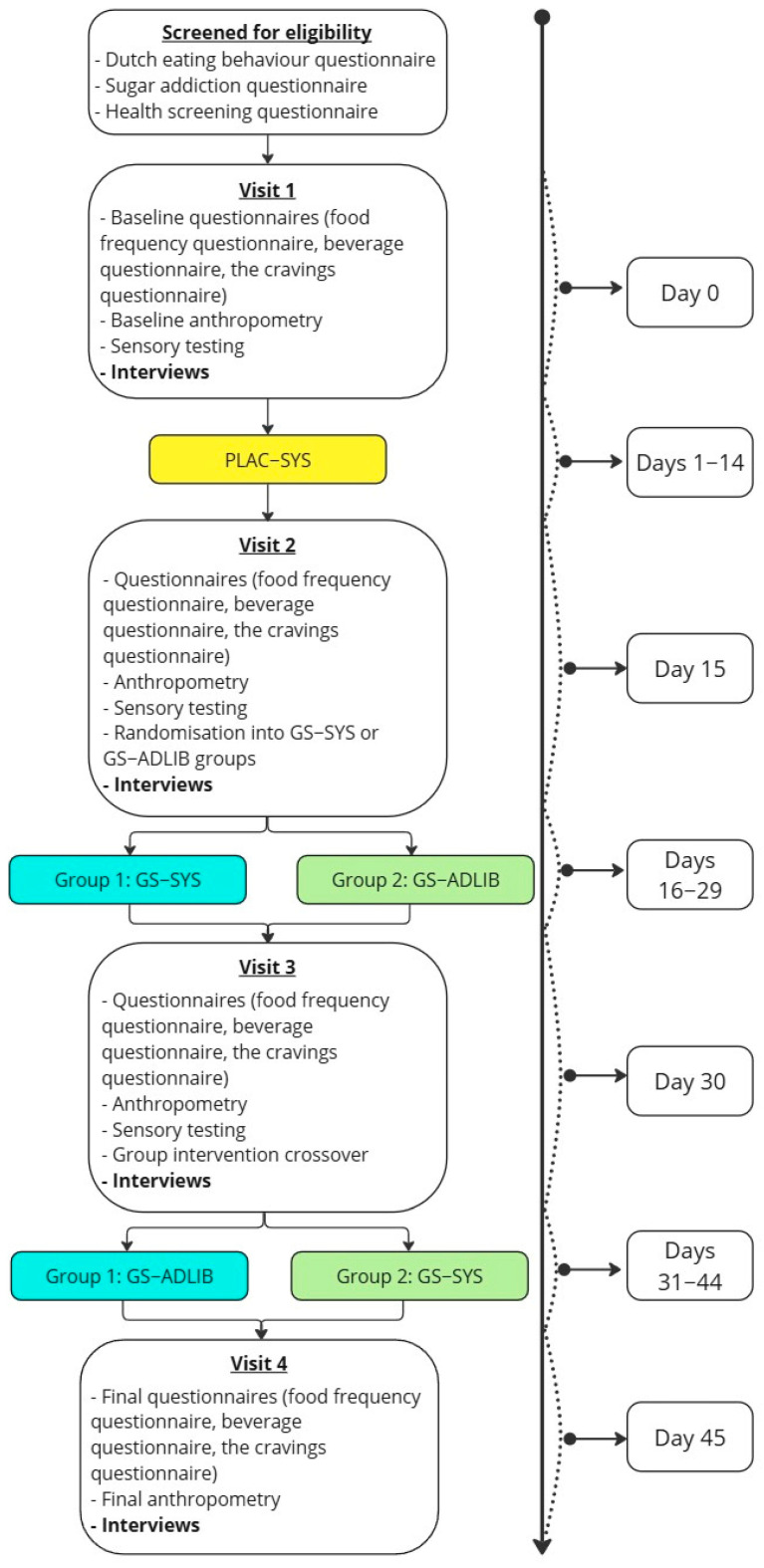
Study design and timeline. All participants were screened for eligibility at the study outset before completing baseline questionnaires, anthropometry, sensory testing and interviews. The study was divided into three 14-day phases, each with a different treatment being administered: PLAC-SYS (placebo systematic)—systematic administration of placebo mint three times a day; GS-ADLIB (Gymnema sylvestre ad libitum)—ad libitum administration of GS mint up to six times a day, at participants’ discretion; GS-SYS (Gymnema sylvestre systematic)–systematic administration of GS mint three times a day. All participants tested PLAC-SYS, followed by randomisation to either GS-SYS or GS-ADLIB and subsequent crossover. After each testing period participants completed questionnaires, anthropometry, sensory testing and interviews.

The study design and process are presented in Figure 1. Participants attended data collection appointments at the Massey University, Auckland campus. Participants underwent data collection at each of these visits, the details of which are not relevant to this study but are described by Hsiao et al. [28]. Details of the 3 arms of the trial are described to provide context for the qualitative exploration. Each 14-day testing period tested one treatment. Participants were led to believe they would be testing three different mints all containing different dosages of GS, and were blinded to the true study protocol. In reality, only two mints were used: one GS-containing mint and a placebo (identical to the GS-containing mints but excluding GS). Both were provided by Nu Brands Inc. (Los Angeles, CA, USA). All participants were treated with the placebo mint for the first 14 days systematically (PLAC-SYS): three times a day at mid-morning, mid-afternoon, and post-dinner. Participants were then randomly allocated to a treatment using a random number generator: systematic GS-treatment (GS-SYS) or ad libitum GS-treatment (GS-ADLIB). Allocated treatments were followed for 14 days, after which each participant crossed over to the other treatment for a final 14 days. The GS-SYS treatment involved taking GS mints in an identical manner to the PLAC-SYS protocol. The GS-ADLIB treatment involved taking GS mints ad libitum, or at the participants’ discretion up to six times a day.

### 2.3. Interview Methodology

Participants completed four 30 min interviews across the study: one at baseline (prior to starting any intervention), and one following each of the 14-day treatment phases (PLAC-SYS, GS-SYS, and GS-ADLIB. Interviews were conducted either in-person (using a digital voice recorder (Sony ICDPX470, Tokyo, Japan)) or using Zoom (Zoom Video Communications, San Jose, CA, USA), depending on participant preference. Interviews were privately conducted with no other individuals present, either virtually or in-person.

Flowchart of study design from recruitment to data collection. PLAC-SYS (placebo systematic)—systematic administration of placebo mint three times a day; GS-ADLIB (Gymnema sylvestre ad libitum)—ad libitum administration of GS mint up to six times a day, at participants’ discretion; GS-SYS (Gymnema sylvestre systematic)–systematic administration of GS mint three times a day.

#### 2.3.1. Interview Guide

The interview guide was developed by IN, RK, and AA with the aim of providing a semi-structured format to the interviews with focused themes, using open-ended questions to encourage participants to share their experiences (see Table S2). Prompts were used as required. The interview guide was reviewed by co-authors and piloted to ensure understanding of questions and appropriateness of prompts. The first author (IN) completed all interviews and took notes during the interviews. No interviews were repeated.

#### 2.3.2. Baseline Interviews

The baseline interview explored participants’ general motivations to eat or avoid SSF, and their perceived level of control over SSF intake, rated from 1 (no control) to 10 (complete control).

#### 2.3.3. Post-Treatment Interviews

Each post-treatment interview (after PLAC-SYS, GS-SYS, and GS-ADLIB, respectively) focused on participants’ experiences with the specific mint used in the prior 14-day period. They were asked to reflect on how the mint affected their motivations to consume or avoid SSF; describe any changes in behaviour, cravings, or awareness of SSF intake; rate their level of control over SSF intake during the past 14 days on a 1–10 scale; and comment on how the mint’s mode of administration (systematic or ad libitum) influenced their experience.

#### 2.3.4. Final Interviews (Post-All Treatments)

In the final interview, participants were asked to evaluate their experience of the full intervention. Specifically, they were prompted to identify their preferred treatment and explain why; indicate which mint they would choose if given a free supply or if required to purchase it; and reflect on any overall changes in their motivations or behaviours related to SSF across the trial.

### 2.4. Data Processing and Analysis

Interview recordings were auto-transcribed from interview software and checked for accuracy by the primary author (IN). All transcripts were de-identified and uploaded to NVivo (QSR International, Burlington,9 Massachusetts) for analysis. Audio recordings were deleted after transcription; transcripts were not returned to participants for comment. Thematic analysis was conducted using an inductive approach, consistent with the interpretive paradigm guiding this study. Coding was carried out by the first author, who began by reading each transcript multiple times to become familiar with the content. Initial codes were generated directly from the data, with attention to patterns and repeated concepts emerging from participants’ experiences and reflections. These codes were then grouped into broader categories that captured commonalities across participants. As themes began to emerge, they were reviewed, refined, and named to reflect the underlying meaning of each grouping. Although the interview guide had identified key areas of interest (e.g., motivation to eat or avoid SSF, perceptions of the treatments), the themes developed from the data rather than being pre-determined.

Once overarching themes were established, NVivo was used to organise the data and explore patterns within and across participant responses. For organisational purposes, each participant’s interviews were grouped into four ‘cases’ representing the four time points: (1) baseline; (2) PLAC-SYS; (3) GS-SYS; and (4) GS-ADLIB. These data should provide information on changes in motivation and self-reported behaviours across the intervention phases.

The final themes and sub-themes reflected both the topics explored in the interview guide and the inductive insights drawn from participants’ narratives. These are presented in Table 1.

## 3. Results

Participants described a range of motivations influencing their consumption or avoidance of SSFs, but in addition they also discussed influences that affected these motivations. They also reported on their opinions on the treatments and commented on their feelings of control of SSF intake with the various treatments. The following sections present the key themes identified in the analysis, supported by illustrative quotes. It should be noted that participants were not asked to provide any feedback on the following findings after they were written up by the authors.

### 3.1. Theme 1—External Influences Affecting SSF Intake

There were many external influences on the participants which were found to significantly affect their motivations to eat SSF. These influences arose from the environment the participants existed in and included factors such as the influence of external environmental factors like occupations and personal responsibilities and the influence of wanting to make a lifestyle change to achieve a better health outcome (e.g., lose body weight or improve health). These external influences were present at baseline, and throughout the study at every treatment phase. There were obviously, as well, the influence of the treatments (PLAC-SYS, GS-SYS, and GS-ADLIB) and the impact of these on the motivation to eat or avoid SSFs which the participants also reported on.

The decision to eat SSF was found to be non-linear, involving external influences on an individual which affect motivations (to eat or avoid SSF) and therefore actual intake of SSF. This process could occur chronologically, with an external influence driving a motivation ultimately deciding SSF intake. The process could also occur in the opposite direction, and may occur in a way where influences or motivations work independently of each other to affect SSF intake.

At baseline, four of seven participants reported being influenced by conscious decisions to reduce SSF intake before even trying any treatment (i.e., they were influenced by lifestyle changes). These originated from desires to improve physical or mental health, or to change body weight. No participant used lifestyle changes to increase their intake of SSF.

*‘I know it’s not good for me. I’m well aware of yeah, just what it is and how much it kind of takes over if you let it take over. I’m very cognitively thinking about everything. I’m like “oh, this does have a bit of sugar in it, probably shouldn’t have eaten that” or “nah, I don’t need that because I don’t need the sugar”.’*

(Participant 3)

Six of the seven participants also felt that their occupations affected their SSF intake at baseline, both independently and through influencing motivation to eat or avoid SSF—these will be discussed in greater detail in 3.2.3 External motivations, as it was shown that not only could the external influences affect motivation, but they could themselves be a motivation to eat or avoid SSF.

After the first 14 days of using PLAC-SYS, all participants reiterated that external influences (e.g., family matters, changes in work, out-of-town trips) significantly affected motivation. Stress often rose, motivating participants to eat out of emotion; or the physical food environments changed (i.e., access to SSF) to shift motivation. In addition, four participants reported that being in the trial itself focusing on SSF intake and health was influencing them to eat less SSF (i.e., classified as a lifestyle change).

*‘If you’re doing a food diary… you have to document what you’re eating. I definitely find that that changes what I eat because I have to start ‘confessing’.’*

(Participant 5)

Again, during the use of GS-SYS all participants identified external influences as both independent influencers of SSF intake, but also influencers on motivation to eat and avoid SSF (see 3.2.3 External motivation for details on the latter). When discussing the effect of external influencers independently on SSF intake, participants reported how these could significantly affect emotions or access to food, which in turn would increase motivation to eat due to hunger or increased availability. Five participants were influenced by lifestyle changes (e.g., being in the trial or weight loss for the summer), which motivated them to eat less SSF.

*‘Cutting down body fat percentage with Summer coming *[motivated me to not eat SSF]. *I want to look better.’*

(Participant 4)

With the use of GS-ADLIB, four participants were motivated to avoid SSFs as part of a lifestyle change due to health or weight concerns, independent of the treatments being used. Only one of these felt the trial itself influenced motivation to eat or avoid SSF. Most participants felt that external influences like occupations and related work trips or vacations were significant drivers of higher and lower intakes of SSFs. There were instances where trips away from home increased or decreased access to SSFs or contributed to stress and emotional eating.

### 3.2. Theme 2—Motivations to Eat or Avoid SSFs

External influences driving a motivation can lead to a decision to consume SSF as a chronological process. The process may also occur in the opposite direction and may occur in a way where influences or motivations work independently of each other to affect SSF intake.

#### 3.2.1. Psychological Motivations

Motivations to eat SSFs were identified by all participants throughout the different phases of the study, with participants eating SSFs out of boredom, stress, emotional triggers, or a general lack of mindfulness around eating. At baseline all participants identified that they ate SSFs due to being bored, out of positive (e.g., celebrating with SSF) or negative (e.g., out of sadness) emotions, or out of a general lack of mindfulness.

*‘There’s been a lot of stress … maybe there’s been a bit of sugar-eating stress relief.’*

(Participant 1)

*‘I’m definitely a boredom eater.’*

(Participant 3)

During the treatment phases, some key motivators for seeking SSFs were psychological (eating out of boredom, stress, emotional triggers, or due to eating mindlessly non-mindfully). However, several participants now described psychological motivation to avoid SSF, with these motivations being attributed to taking the treatments (both PLAC and GS treatments). Two participants thought PLAC-SYS decreased motivation to eat SSF because it promoted mindfulness, and one of these even felt they appreciated savoury flavours more with administration of PLAC-SYS.

*‘I didn’t have as much as I’d normally have. I was able to probably stop myself after a few* [servings]*, be like, ‘aw, I’ve had enough’.’*

(Participant 4)

*‘I looked at the sweet chili sauce… I’m actually eating a lot more sugar than what I think I’m eating because all of these extras. And then I took* [PLAC-SYS] … *the sweet chili sauce wasn’t as pleasurable* … *and then the next day, I thought, ‘you know what, I don’t actually need that sweet chili sauce at all’. So, I had* [PLAC-SYS]*, didn’t have sweet chili sauce, just had the quiche and I was fine.’*

(Participant 7)

Two participants also reported GS-SYS directly increased motivation to avoid SSF due to an increased awareness of food intake (i.e., mindfulness). The treatment was taken as prescribed and when the anti-sweet effects elapsed, participants consider if they still wanted SSFs. This strategy allowed cravings to pass, and decreased the volume of SSF consumed.

*‘You also know that the food you eat after it won’t be quite as satisfying. So, you’re like, OK, this is OK. I can wait or I cannot* [have any SSFs] *at all.’*

(Participant 6)

However, GS-ADLIB was overall identified more as driving psychological motivation to avoid SSF. Four of the seven participants discussed how GS-ADLIB psychologically motivated them to avoid SSFs by helping them assess their true want for SSFs (i.e., increased mindfulness).

*‘It was almost like a self-test. … How badly do I want this sweet thing? Like, you know, am I prepared to have* [GS-ADLIB]*, and then make a specific trip back downstairs in an hour’s time to have* [SSF]*? … so, it was a little bit like a test.’*

(Participant 1)

*‘In some ways, it’s kind of helped me … learn to not give into the craving straight away. … It does sort of give me that time to go through that process; you know, not be so reactive.’*

(Participant 5)

Overall, psychological motivations to eat SSFs—such as boredom, stress, and emotional triggers—were commonly identified throughout the study; and during the intervention phases, several participants described a shift toward greater mindfulness and reflective decision-making, particularly when using GS-ADLIB.

#### 3.2.2. Habitual Motivations

Habits were another common driver of SSF intake. Many participants (e.g., four at baseline) identified routine consumption of SSFs as part of their daily lives, such as dessert after dinner or specific snacks with tea or coffee.

*‘It’s all around… habit probably. I’ve always had a cup of tea and a biscuit, so it’s my go-to. Growing up was dessert after dinner, biscuits for morning and afternoon tea.’*

(Participant 1)

These habitual patterns were often automatic and sometimes embedded in social settings. Some participants realised the depth of these habits only when their behaviour was interrupted by the treatment phases.

*‘*[My colleagues] *actually noticed because when we went to the I, I always order the blueberry muffin with morning coffee. And they were like, “blueberry muffin?” and I was like, “no, not today”. That’s how much of in a routine* [I was in] *that people I work with, and see once a month, know that I always have a blueberry muffin. And I was like, wow, that’s quite a bad habit to me.’*

(Participant 7)

Two participants felt that PLAC-SYS helped break habitual intake of SSFs. The GS-SYS treatment phase also disrupted habitual SSFs consumption for two participants, with the act of taking GS-SYS at regular times serving as a behavioural cue to break the pattern of mindless eating.

*‘What’s that um experiment, or the study or they’d ring the bell, and it would make the dog salivate or whatever? Pavlov’s dog, yeah. That’s me with* [GS-SYS]*. Eat* [GS-SYS]*, and then that’s it, times up.’*

(Participant 2)

Using GS systematically (i.e., GS-SYS) drove habitual motivation to avoid SSF more than GS-ADLIB, as no participant discussed ad libitum GS administration as a way to help interrupt the habitual intake of SSF. Several participants described habitual eating being replaced with more conscious decisions, suggesting that systematic administration of a treatment (PLAC-SYS or GS-SYS) had potential to interrupt automatic consumption patterns.

#### 3.2.3. External Motivations

All participants described external factors—such as work schedules, holidays, access to food, and social environments—as key influences on their motivations to consume SSFs throughout the study. These factors could either increase or decrease SSF intake, depending on the situation. In particular, occupations (i.e., working or studying) were significant drivers to eating or avoiding SSF, with six of the seven participants identifying these are influencers on SSF intake. For one participant working fulltime, sometimes there was less motivation, other times (for instance during meetings where SSFs were available) there was increased temptation to eat SSFs because they were easily accessible.

*‘If I get busy, and I haven’t had like, my next meal that I usually would have; like a lunch whatever, that can usually mean that I’ll just… eat anything.’*

(Participant 2)

*‘I suppose, the least* [I] *want to eat it is actually just because I’m busy.’*

(Participant 7)

After testing PLAC-SYS, all participants reiterated that external influences (e.g., family matters, changes in work, out-of-town trips) significantly affected motivations to eat or avoid SSFs. Again, external factors could increase or decrease SSF intake depending on the context; for example, one participant, due to being on holiday away from home, was actually out of a high-SSF environment and as such found there was less motivation to eat SSF.

*‘I did go away for a week. So, I wasn’t around roommates offering me* [SSFs]*.’*

(Participant 4)

During testing of the GS treatments, participants’ external influences significantly affected emotions or access to food, which in turn would increase motivations to eat due to hunger or increased availability.

*‘Where I work, there’s lots of lovely, yummy goodies, whereas this week being at* [my second job]*, I have occasionally had, like, a little biscuit with my cup of tea. But the access is a lot more limited because you’re with people all the time.’*

(Participant 6)

The effect of external factors sometimes overrode any influence of the GS treatments. For example, some participants noted that being on holiday diminished their motivation to use GS mints as the desire to enjoy treats took precedence.

*“I went on holiday for a few days, had some alcohol and desserts. I wanted to enjoy the holiday, so I didn’t worry about taking* [GS-ADLIB] *or avoiding* [SSF] *for those days.”*

(Participant 3)

External influences—particularly work demands, social settings, and holidays—played a significant and sometimes overriding role in participants’ motivations to eat or avoid SSFs, with context often determining whether GS treatments were used consistently or set aside.

#### 3.2.4. Pleasure-Seeking Motivations

Pleasure and rewards were central to many participants’ motivations for consuming SSFs, with the most identified motivation to eat SSF across the study being pleasure-seeking-related. At baseline, all participants wanted to eat SSFs because they tasted good and described enjoying sweet foods purely for the taste, or as a source of gratification or indulgence.

*‘Obviously like most people I end up eating more than you’d want to, it’s probably a bit of endorphins. The brain knows it tastes good, it gives you energy, once that you have it.’*

(Participant 4)

*‘I do sort of naturally have a sweet tooth. I will intentionally not have sweet foods in the house. If it’s there I will eat it.’*

(Participant 5)

These pleasure-driven motivations were strong and persistent during the intervention phases as well, with all participants during testing of PLAC-SYS, GS-SYS, and GS-ADLIB being motivated to eat SSFs because of perceived palatability. During testing of PLAC-SYS six of the seven participants reported changes to motivations to avoid SSFs, reporting that PLAC-SYS motivated avoidance as using it made SSF taste unpleasant. This was directly attributed to CON-SYS by producing a minty after-taste, making SSFs unpalatable, being sufficiently sweet to satiate SSF desire, or reducing cravings for SSF directly.

*‘It’s just that taste was so strong in my mouth, definitely it did have a negative effect. I haven’t had big cravings, and then whenever I do I just have* [PLAC-SYS] *and an hour later I don’t have* [them] *anymore.’*

(Participant 4)

Similarly, six of the seven participants also reported GS-SYS affected motivations to eat SSFs due reduced pleasure. Some participants experienced mild taste changes; for others it was severe. Three participants also felt that GS-SYS reduced cravings for SSFs.

*‘I did test out having different foods with* [GS-SYS]*, and it was awful. So, I think I had like, a piece of chocolate once, and I was like, this is so gross.’*

(Participant 2)

There were three participants who found taking GS-SYS affected SSFs even outside of the timeframe they were administered, making SSFs excessively sweet and therefore unpleasurable.

*‘It’s definitely given me an appreciation for how sweet some things are. Like, to have something that you normally have, and then take all the sensation of sugar out of it, and then bring it back in, it’s like, whoa. Like, sometimes that’s quite a lot, almost to the point where you’re like, oh, this makes me feel a bit a bit peaky.’*

(Participant 6)

However, notably, one participant manipulated timing of SSF consumption around the time when the effects of the treatment had elapsed, so that the foods were still pleasurable. Although the unpleasurable tase did initially influence SSF intake, it was not enough to affect motivations to eat SSF and therefore actual intake of food.

*‘It didn’t have a massive effect on my intake of sugary foods … I would just wait for the time frame of* [GS-SYS] *to wear off, then I’d have something sweet… so overall it didn’t affect how or what I ate.’*

(Participant 5)

#### 3.2.5. Physiological Motivations

Physiological motivations—such as hunger, energy needs, weight management, and health concerns—were commonly cited reasons (at baseline and during test of GS-SYS five participants reported avoiding SSFs for physiological reasons) for SSF consumption or avoidance throughout the study.

*‘Obviously when I cut* [weight] *I look a lot better than a normal maintenance* [weight]*. I keep that in mind.’*

(Participant 4)

*‘I guess weight gain would probably be my main* [motivation to not consume SSF] *… I think it’s just the fear of: if I keep eating this, I’m going to get really fat.’*

(Participant 5)

Sugar-sweetened foods were also viewed as a quick source of fuel that could be utilised quickly during a busy day by two participants at baseline.

*‘You’re like “oh god, I still have three hours left of my day” …It’s probably then* [I feel like eating SSF the most]*. It’s especially during exam time. It’s when your brain is working so hard and you’re so hungry all the time. It’s like, “yeah, let’s just top up with some sugar”.’*

(Participant 6)

In addition, four participants also identified that prior knowledge of the effect of SSFs on health motivated them to avoid SSFs. This was partly originating from social factors for one participant, or prior knowledge due to personal or occupational studies for all.

*‘My mum thinks carbohydrates are the devil. And so, in my house, we never had dessert, we hardly ever had bread and things like that so like, I think I was always aware that I shouldn’t have too much sweet things.’*

(Participant 6)

The use of the treatments (PLAC-SYS or GS treatments) was not reported as directly influencing these motivations for any participants.

### 3.3. Themes 3 and 4—Opinions on Each Treatment Arm: Value of Mints

Six participants liked PLAC-SYS and reported it had a similar flavour to commercially available mints. One participant experienced mild nausea when taking it on an empty stomach while another expressed ambivalence about the long-term health implications. None of the participants wanted a free sample of PLAC-SYS.

Four of the seven participants also reported liking GS-SYS, finding the treatment tasted better than GS-ADLIB (despite the treatments being identical). It was overall more effective than PLAC-SYS because it reduced motivations and cravings for, and total intake of, SSFs. Dislikes of GS-SYS were mostly related to its unpleasant taste, texture, and the way it altered the taste of SSF. Two participants wanted a free sample of GS-SYS.

Participants were deceived into believing they were trying three different GS-containing mints with different dosages of GS. However, even with this deception it was the systematic administration of the mint, rather than any perceived different dosage, that was notably preferred by most participants. Participants noted they would pay for GS-SYS if it were commercially available (dependent on price and their income). Three participants preferred GS-ADLIB to GS-SYS; feeling it was more effective at making SSF less desirable and reducing their intake. Negative opinions were related to the GS-ADLIB treatment’s taste, making SSF taste unpleasant, or an unpleasant after-taste.

*‘It was good at suppressing the ‘want’ for sugary food.* [GS-ADLIB] *itself is ok, but it made food taste bad.’*

(Participant 3)

Three participants would have accepted GS-ADLIB for free, and only one would pay for the treatment. Others felt their sweet tooth was not significant enough to justify paying for GS-ADLIB. When compared to participants’ opinions on PLAC-SYS, both GS-SYS and GS-ADLIB were more effective at making SSF unpalatable, and thus they preferred the GS treatments over PLAC-SYS.

### 3.4. Theme 5—Changes in Level of Control over SSF Intake

Baseline control levels over SSF intake varied. Three participants reported a level of 7 or 8 and felt disciplined about their baseline intakes. Three described their control levels on a sliding scale, with daily variation.

*‘Majority of the time I’d say I’m pretty in control. But … there’s a moment where there’s zero control;* [I] *just eat whatever is in front of me.’*

(Participant 4)

Three participants reported no change in control level, whilst four felt their control level had increased directly because of PLAC-SYS. Two participants felt their control level decreased during testing of GS-SYS compared to baseline (possibly because of increased stress and hunger). Two participants felt their control was higher than baseline while testing GS-SYS; however, one felt it was higher while testing GS-SYS and the other felt it was lower than when testing PLAC-SYS, possibly because of external influences. During testing of GS-ADLIB, external factors contributed to changes in control for three participants. Vacationing made two participants feel less controlled; whilst finishing a semester of tertiary education resulted in higher control for another, due to spending less time in environments where SSFs were readily accessible.

*‘This time it was one hundred percent: I was making all the decisions about the sugar I was taking.’*

(Participant 7)

## 4. Discussion

### 4.1. Summary of Key Findings

This study found that in a sample of healthy adults who had a sweet tooth: (1) both GS treatments increased motivations to avoid SSFs due to increased mindfulness and decreased palatability of SSFs; (2) that motivations were also affected by external influences; (3) that GS-ADLIB was the most successful treatment for reducing intake of SSF; (4) that participants’ level of control over SSF intake varied throughout the trial.

### 4.2. Mindfulness and Behaviour Change

Motivation to avoid SSF was higher with GS treatments due to SSF being less palatable or enjoyable, which made them consciously decide that SSF intake was unnecessary. Both groups reported an increase in mindful eating, which involves using physiological cues rather than emotions to dictate food choice, which is a technique that has been used for treating eating disorders and obesity [34,35]. Because GS negatively affects pleasure from SSFs for 30–120 min, participants would heed satiety cues better during this time [36]. After effects of GS elapsed, participants may have decided SSFs were not needed after all, despite initial high motivation to eat them. An additional aspect of being mindful (not just in relation to eating) is that many of its fundamentals are also foundations of the initial phases of the Transtheoretical Model, or Stages of Change Model, which is used to assess inclination for behaviour change [37,38]. The second stage of ‘contemplation’ involves conscious tuning to long-term goals to transition to subsequent stages [37]. For participants in this study to achieve lower SSF intakes they likely used mindful eating to execute beneficial actions classically seen only in the fourth stage of ‘action’. Two participants also found using the PLAC-SYS increased their motivation to avoid SSF because of enhanced mindfulness. This indicates that mindful eating, with or without GS, could help reduce motivation to eat SSF. Mindful eating has had varying effects on SSF intake and has been linked to reduced confectionary intake and lower fasting blood glucose in obese adults over 12 months [35,39]. Short-term restrictive diets for weight loss are ineffective at changing body weight long-term, let alone producing long-term maintenance to low-sugar diets, which is one reason mindful eating may be a preferable intervention for long-term dietary change [40,41].

### 4.3. Gymnema Sylvestre and Taste-Driven Motivation

Participants also felt GS changed motivations by making SSFs taste unpleasant. Previous research found that GS significantly reduced pleasure derived from SSF [16,20,21,22,23,24]. Most of these studies also found GS reduced SSF intake [16,20,21,22,24]. Overall, research on relationships between having a sweet tooth and non-communicable disease development is limited. However, hypothetically, this group would benefit from interventions targeting SSF preferences as they have higher desire for these foods which are already associated with non-communicable disease risk [17]. Turner et al. and Turner et al. found that GS reduced pleasantness of and desire for chocolate to a greater degree in participants that had a sweet tooth compared to those that did not [16,22]. The present study supports these findings and provides novel evidence that the reason GS is more effective in those with a sweet tooth may be because the herb increases motivation to avoid SSF. Although GS reduced immediate motivation to eat SSFs due to reducing their palatability, some counteracted this problem by waiting for the effects of GS to elapse before eating SSFs or eating them at a different time of day. This points to the desire for SSFs overriding attempts to change behaviour. The stages of change model posits that individuals are only truly motivated to change behaviour in the fourth stage of ‘action’, when they are receptive to receiving and seeking support [37]. Further research into whether GS can provide a synergistic effect with mindfulness to help individuals move through stages of behavioural change successfully, is merited.

### 4.4. Influence of External Factors

External factors were found to significantly affect motivation to eat SSFs amongst the participants in this study, including individual experiences, circumstances, and environments, similar to those in previous studies [42,43]. ‘Food addiction’ is controversial and not recognised by *The Diagnostic and Statistical Manual of Mental Disorders* (DSM-5) [44,45]. Findings from drug- and alcohol-related research may therefore not be applicable to food overconsumption, which is not synonymous with addiction [46]. Turner et al. likened nicotine patches for smoking cessation to using GS for SSF reduction, and proposed that when used over 14 days, GS can be used as a short-term tool (in addition to other behavioural modification programmes) to reduce habitual SSF intake and build awareness around SSF intakes [22]. The current study supports this hypothesis by showing that GS can be used to reduce SSF intake over 14 days through motivational change.

### 4.5. Treatment Preferences and Acceptability

The third main finding was that GS-SYS and GS-ADLIB were not only preferred more by participants than PLAC-SYS, but they were also reportedly more effective. Medication adherence may be lower if they are perceived negatively [47]. Preference of the GS treatments indicate they may be perceived as effective interventions for reducing SSF intake by this study’s participants. Participants, in general, found GS-ADLIB a more successful treatment than GS-SYS. Individual preference of treatment administration may be a contributing factor as to why a clear preference was unindicated, possibly more so than the perceived dosages of the treatments [47].

### 4.6. Self-Control and Intervention Design

The fourth main finding was the report of varied control levels over SSF intake across the study. Most participants did not feel control levels changed during the testing of PLAC-SYS, nor GS-SYS. The biggest changes were seen during the testing of GS-ADLIB, which may be attributable to external factors as well as GS-ADLIB. Self-control has been shown to significantly affect morbidity and mortality, and higher levels have been previously associated with higher intakes of fruits and vegetables, fewer instances of overeating, and being less likely to engage in behaviours that cause weight gain [48,49,50,51]. Participants in the current study reported stress from external factors drove their motivation to eat SSF and lowered their self-control. Lifestyle interventions designed to improve dietary quality (e.g., lower SSF intake) should consider individual stressors and how these might override interventions, no matter how effective the interventions are. This study supports previous evidence indicating that GS does reduce intake of SSF, but it also highlights that external factors were contributors to participant self-control over SSF intake [16,20,21,22,24,25,52]. As GS-ADLIB seemed to contribute to higher self-control, and similarly external factors seemed to be strong determinants of self-control, our study supports the proposal by Turner et al. that GS may be beneficial as part of larger lifestyle interventions, rather than used in isolation [22]. Additional measures (e.g., dietitian support, nutrition counselling, coaching) may be required in addition to GS to address these external factors and help increase the efficacy of GS treatments.

### 4.7. Strengths and Limitations

A strength of this study was the in-depth analysis into the effects of GS intake on motivation to eat SSFs using qualitative methods. Second, the health screening questionnaire was extremely detailed and excluded participants for an exhaustive number of health reasons. None of the participants had altered smell or taste perceptions, therefore not impacting on the consistency of responses to GS treatments. During the interview process, the first author (IN) had no pre-existing relationship with any of the study participants and endeavoured to remain neutral throughout interviews. She was a student dietitian at the time of data collection, and her student status may have encouraged trustworthiness and openness in participants during interviews. Participants were not informed of the interviewer’s personal goals or reasons for conducting the research, and no relationship was established prior to participation. No characteristics, biases, or assumptions were disclosed to participants during the study. On completion of the interviews, a software transcription service transcribed the audio recordings. The primary researcher (IN) then reviewed each transcript for accuracy against the recordings in full. As the interviewer, IN was able to verify the transcription quality and correct errors. Review by a second researcher would have further strengthened transcription reliability and reduced the risk of undetected errors.

This study was limited by the screening questionnaires not assessing whether participants were already undergoing or have an interest in lifestyle interventions, such as weight loss initiatives, prior to recruitment. These individuals may have had differing motivations to other study participants. Another limitation was the small number of participants that agreed to the interviews conducted throughout the study, mainly due to the high burden of the randomised cross-over trial requiring interviews across all three study phases. As a result of this small sample, saturation may not have been achieved across all themes. It is also possible that the participants who volunteered for interviews were more motivated or reflective than the broader trial population, introducing potential bias. Accordingly, findings from this sub-study may not fully represent the wider experiences of all trial participants. However, consistent findings across the 28 interviews suggest that a study using the same protocol with a larger sample would be valuable. Future research could explore potential gender-based differences in response to GS administration and motivation to eat SSF. In the present study, two of the seven participants were male and five were female, which, combined with the small sample size, prevented meaningful gender-based analysis and limited the scope of the conclusions.

The extent to which participants were actually mindfully eating (and changes to this across the study) was not directly measured. Seeing as participants notably identified the effect of the GS treatments on their ability to eat mindfully, it would be important to measure mindful eating in future studies investigating effect of GS intake on motivations to eat SSFs. Finally, participant age range was wide in this study (18–60 years) which may have introduced variation in sensory perception or behavioural responses to GS treatment. Age-related differences in taste perception, as well as possible food preferences and motivations to eat SSFs, could have influenced how participants experienced the interventions.

## 5. Conclusions

The main findings of this study were (1) both GS-SYS and GS-ADLIB increased motivation (more than PLAC-SYS) to avoid SSF because the GS treatments increased mindful eating and decreased pleasure from eating SSF; (2) external influences affected both motivation as well as GS intake; (3) GS-ADLIB was deemed the most successful treatment; (4) the level of control participants felt over SSF intake fluctuated across the study due to both external factors and the use of the different treatments. By promoting mindful eating patterns, using GS might be beneficial, especially when used as part of interventions for reducing SSF intake. In addition, the preference of GS over the placebo indicated that it has potential to be positively perceived and therefore be a well-adhered-to treatment. Motivations for eating SSFs were highly complex, and consumption was affected by not only external influences, but also levels of self-control. Behaviour change requires a multidisciplinary approach, and current interventions for reducing sugar intake may not target these complex motivations appropriately. This study provides novel evidence that adults with a sweet tooth may experience motivation changes related to SSF consumption when using either GS-SYS or GS-ADLIB, but in particular the GS-ADLIB treatment approach was deemed most successful.

The findings from this qualitative sub-study corroborate results from the larger randomised controlled trial, which demonstrated that GS administration reduced SSF intake in adults [28]. Together, these findings suggest that GS may be used as part of interventions to reduce SSF intake, not only by taste alteration, but also by promoting mindfulness of hunger and satiety cues. This study provides preliminary qualitative evidence that GS can influence motivation to avoid SSF in adults who identify as having a sweet tooth. However, the small sample size and potential for selection bias (e.g., more motivated participants agreeing to interviews) limit generalisability. Further research using a larger sample with a narrower age range, which explores gender-based responses to GS, is needed to confirm these findings and assess the applicability of GS within dietary interventions targeting SSF intake.

## Figures and Tables

**Table 1:** Thematic analysis and theme construction from twenty-eight interviews.

Child Codes	Parent Codes	Quotes	Themes
External influences related to occupations, daily activities and responsibilities	External influences affecting SSF intake	*‘I think I’ve had a placebo this week, and it’s done nothing.’ (Participant 1)* [With GS-ADLIB I would forget] *to take it and being like well, “*[I] *don’t really feel like taking it now,” so didn’t have as much as an effect on me. Whereas like with* [GS-SYS]*, I’ll take it because it was like scheduled.’ (Participant 2)* *‘Probably just making smarter decisions when it comes to eating? I know I can, I’ve done enough study behind it to know what I should and shouldn’t be eating.’ (Participant 3)* *‘If I say* [compare the] *corporate rush mode of conferences and workshops* …*, that’s a very different space to be in than to be working from home in a relaxed environment and where you can control a whole lot of things. So I do think lifestyle has an impact on it.’ (Participant 7)*	External influences affecting SSF intake
External influences related to lifestyle changes			
External influences related to influence of PLAC-SYS, GS-SYS and GS-ADLIB			
Mindfulness and awareness	Psychological motivations	*‘I’m thinking more about… not intuitive eating, but sort of thinking like, do I? Do I need a biscuit, am I actually hungry? So I’ve been trying to do a little bit more about that. But then sometimes I go, yeah I do. I really do want this.’ (Participant 1)* *‘Across that lunch time* [I’m] *not really big on sugar* … *just because I’m busy. I suppose you’re busy with other things so you just don’t think about it.’ (Participant 7)*	Motivations to eat or avoid SSFs
Emotions			
Boredom			
Knowledge	External motivations	*‘I really like sweet things a lot, I could eat a whole meal of sweet things to be fair, but it’s one of those things where I just know that it’s not beneficial. There’s not a lot of protein in those sweet things. There’s not a lot of like fibre and stuff like that. It’s just not the best. So, I’m aware that it’s not good for me.’ (Participant 2)* *‘I didn’t really eat sugar this past week. I did go away for a few weeks. So, I wasn’t around roommates offering me* [SSF]*.’ (Participant 4)* *‘My mum thinks carbohydrates are the devil. And so, in my house, we never had dessert, we hardly ever had bread and things like that so like, I think I was always aware that I shouldn’t have too much sweet things. But now I live on my own.’ (Participant 6)*	
Finance			
Environment and access			
Social factors			
Habits	Habit-related motivations	*‘*[My colleagues] *actually noticed because when we went to the I, I always order the blueberry muffin with morning coffee. And they were like, “blueberry muffin?” and I was like, “no, not today”. That’s how much of in a routine* [I was in] *that people I work with, and see once a month, know that I always have a blueberry muffin. And I was like, wow, that’s quite a bad habit to me.’ (Participant 7)*	
Wanting	Pleasure-seeking behavioural motivations	*‘There’s always room for dessert. If I go out for dinner, I have to look at the dessert menu before I order my mains.’ (Participant 1)* *‘The mint is not sort of addressing the underlying issue* [which] *is that I’m craving something sweet, and then I want to taste the sweet thing. So, by taking the mint I’m actively deciding to make that sweet thing taste not sweet. So, I might as well just have a carrot.’ (Participant 5)* *‘I’m currently in the right phase of my* [menstrual] *cycle for that* [sweet] *craving to be around.’ (Participant 6)*	
Pleasure and reward			
Hormone and cravings			
Health	Physiological motivations	*‘I don’t eat a lot, especially for my body type being so active. I’ll have dinner, but after the dinner I might still* [be hungry] *because I haven’t eaten enough, so I guess it kind of falls into that, too. It’s like, I need to eat something. Again, I just want something sweet, so I’ll have chocolate.’ (Participant 3)* *‘I know what calories I consume* [in] *a day. I keep on top of my physique and training. So, instead of having a nutritious meal for* [a certain amount of] *calories* … *I have the same calories in a dessert* … *I’m not getting proper nutrition. It’s more knowing that the nutrition you could’ve had in that meal would’ve helped you [with fitness or physique goals].’ (Participant 4)* *‘I guess weight gain would probably be my main* [motivation to not consume SSF] … *my weight doesn’t really fluctuate. I think it’s just the fear of if I keep eating this, I’m going to get really fat.’ (Participant 5)*	
Hunger, satiety and energy			
Weight			
Negative opinions on mints	Opinions on mints	*‘There is nothing to like about* [GS-ADLIB]*. It just took all the pleasure, all the hedonistic part of having that sweet thing away… So that good feeling that you got from it—that is the point of eating something sweet -was just not there, so there was no point* [in eating SSF]*’. (Participant 1)* *‘*[GS-ADLIB] *was good at suppressing the “want” for sugary food. The mint itself is ok but made food taste bad.’ (Participant 3)* *‘*[I] *can’t really tell that big of a difference between* [GS-ADLIB and previous mints]*.’ (Participant 4)*	Opinions on treatment arms
Positive opinions on mints			
Mixed opinions on mints			
Which mint would participants accept for free	Value of mints	*‘Probably* [GS-ADLIB]*. I feel like maybe I could find a way to make it work. I think the thing with* [PLAC-SYS] *is that yeah, because it tasted nice, I would sometimes have it instead of the sweet food I kind of decided that that was better just to have a small mint.’ (Participant 5)* *‘Everyone goes out and buys paracetamol. Everyone has paracetamol. It’s nice having the option. I can see with the benefit lies in people who feel completely out of control all the time.’ (Participant 6)*	
Which mint would participants pay for			
Feeling more in control over SSF intake	Changes in level of control over SSF intake	*‘I think* [GS-SYS is] *good for that, and it can help you just have a bit more control over things.’ (Participant 2)* *‘There’s a moment where there’s zero control* [over SSF intake]*; just eat whatever is in front of me. So sometimes I could go a couple of weeks of two thousand calories a day, and then I have six thousand in the seventh* [day]. *You don’t really think. You’re just grabbing and eating. Bit animalistic.’ (Participant 4)*	Changes in level of control over SSF intake
Feeling less in control over SSF intake			

## Data Availability

The original contributions presented in this study are included in the article/Supplementary Material. Further inquiries can be directed to the corresponding author.

## Supplementary Materials

The following supporting information can be downloaded at: https://www.mdpi.com/article/10.3390/nu17172718/s1, Table S1: COREQ checklist; Table S2: Interview guide. Ref. [53] is cited in Supplementary Materials.

## Abbreviations

The following abbreviations are used in this manuscript:

GS	Gymnema sylvestre
GAs	Gymnemic acids
PLAC	Placebo
SYS	Systematic trial
AD-LIB	Ad libitum trial
SSF	Sugar-sweetened food
