# Supplementary material for: The Effect of Gymnema Sylvestre on Motivation to Consume Sweet Foods—A Qualitative Investigation
Source: Nutrients. 2025 Aug 22;17(17):2718. doi: 10.3390/nu17172718 (PMC12430398; doi:10.3390/nu17172718)
Supplement: Supplementary file 1 [file nutrients-17-02718-s001.zip › Table S2. Interview guides.pdf]

Table S2. Interview Guide. Guide used during initial, second, third and final interviews.

Research questions

- What is the effect of GS-containing mints on motivations to consume sugar-sweetened food?
- What is the effect of GS-containing mints on the desire to consume sugar-sweetened food?
- What is the effect of GS-containing mints on the total intake of sugar-sweetened food in people who self-identify as having a sweet tooth?

Focus Area	Questions
Introduction<br>Ice breaker / warm - up	● Thank you for coming back in today.<br>● Ice breaker<br>● Asked in **initial interview** only:<br>○ Thank you for volunteering<br>○ Purpose of interviews: in-depth information about their motivation to consume sugar food intake<br>○ Initial ice breaker: “how did you find out about our study?”<br>○ Tell me a bit about why you decided to volunteer for this study.<br>○ What’s your favourite sugary food to eat?
How participants are finding the study?	● Asked in **second, third and final interviews** only:<br>● How have you found these past two weeks in the trial?<br>● Are there any parts of the trial you’ve found difficult?
Consumption of sugar-sweetened food	● Asked in **initial interview**: Tell me about your relationship with sugar sweetened foods right now.<br>● Asked in **second, third and final interviews**: Tell me about how you’ve been finding sugar sweetened foods in the past two weeks.
Motivations	● Asked in **initial interview** only<br>○ “One really important part of this study is looking at the motivations to consume sweet food…”<br>○ What would you say is your biggest motivator to eat sugar sweetened food?<br>○ If asked what the interviewer means… “these are any foods that contain added sugar, so it would include things like coffee and sugar, cake, sugar sweetened beverages, sauces with sugar etc.”<br>○ How did you come to that decision?<br>○ What does the phrase, “dessert stomach” mean to you?<br>○ Do sugar sweetened foods bring you pleasure?<br>○ We spoke just before about how you felt your motivations to eat sugar food were _______. What do you think would change these motivations?<br>○ What would motivate you to not consume sugar sweetened foods?<br>● **Asked in second**, third and final interviews only:

<table>
<tr><td></td><td>

Considering how you've been using the mint in the past two weeks, what would you say is your biggest motivator to eat sugar sweetened food?
How did you come to that decision?
How have you been finding the effects of the mint on what you eat?
Have you thought any more about what motivates you to not eat sugar sweetened food?

Asked in final interviews only (in addition to previously listed bullets):

You've now used a few different kinds of mints. Tell me about how you found each of them.
What would you say your biggest motivators to eat sugar sweetened foods are now at the end of the study?
How did you come to that decision?
What would you say are your biggest motivators now to NOT eat sugar sweetened foods?

</td></tr>
<tr><td>Desire</td><td>
Asked in initial interview only:

What does having a "sweet tooth" mean for you?
When would you say you most feel like eating sugar sweetened foods?
And when would you say you feel least like eating sugar sweetened foods?
On a scale of 1-10, with 1 being no control at all and 10 being complete control, how in control do you feel over your consumption of sugar sweetened foods?

Asked in second, third and final interviews only:

How has your "sweet tooth" been in these past two weeks?
When would you say you most feel like eating sugar sweetened foods at the moment?
Probing tools / questions to elaborate.

Tell me a bit about that.
What makes you say that?
Silence

And when would you say you felt least like eating sugar sweetened foods these past two weeks?
How in control do you feel over your sugar consumption?

</td></tr>
</table>
